# House Cricket (*Acheta domesticus*) Meal as an Alternative Protein Source for Fattening Rabbits: Effects on Growth Performance, Nutrient Digestibility, Intestinal Morphology, and Histopathology

**DOI:** 10.3390/biology15201898

**Published:** 2026-10-21

**Authors:** Gabriele Brecchia, Nour Elhouda Fehri, Albana Munga, Giacomo Rossi, Giulio Curone, Enxhi Hasani, Livio Galosi, Juan José Pascual, Francesca Falcinelli, Lucia Biagini, Marta Castrica, Daniele Vigo, Olimpia Barbato, Xhelil Koleci, Laura Menchetti, Alda Quattrone

**Affiliations:** 1 Department of Veterinary Medicine, University of Perugia, Via San Costanzo 4, 06126 Perugia, Italy; gabriele.brecchia@unipg.it (G.B.); francesca.falcinelli@collaboratori.unipg.it (F.F.); olimpia.barbato@unipg.it (O.B.); 2 Department of Veterinary Medicine and Animal Sciences, University of Milan, Via dell’Università 6, 26900 Lodi, Italy; nour.fehri@unimi.it (N.E.F.); giulio.curone@unimi.it (G.C.); daniele.vigo@unimi.it (D.V.); 3 Faculty of Veterinary Medicine, Agricultural University of Tirana, Koder Kamez, 1029 Tirana, Albania; amunga@ubt.edu.al (A.M.); enxhi.hasani@umb.edu.al (E.H.); xhelil.koleci@ubt.edu.al (X.K.); 4 School of Biosciences and Veterinary Medicine, University of Camerino, Via Circonvallazione 93/95, 62024 Matelica, Italy; giacomo.rossi@unicam.it (G.R.); livio.galosi@unicam.it (L.G.); lucia.biagini@unicam.it (L.B.); laura.menchetti@unicam.it (L.M.); 5 Department of General Training and Vocational Development, Faculty of Medical Sciences, Barleti University, Rruga Frang Bardhi, Selitë, 1046 Tirana, Albania; 6 Institute for Animal Science and Technology, Universitat Politècnica de València, Camino de Vera s/n, 46022 Valencia, Spain; jupascu@upv.edu.es; 7 Department of Life Science, Health and Health Professions, Università Degli Studi “ Link Campus University”, Via del Casale di S. Pio V 44, 00165 Rome, Italy; m.castrica@unilink.it

**Keywords:** *Acheta domesticus*, cricket meal, edible insects, rabbit nutrition, fattening rabbits, sustainable feed, alternative protein sources, digestibility, insect meal

## Abstract

House cricket (*Acheta domesticus*) meal is a promising alternative protein source, but evidence regarding its nutritional value and effects in rabbits remains limited. This study aimed to (i) evaluate the effects of replacing part of the soybean meal with 3% *A. domesticus* meal on growth performance and intestinal and hepatic histology in growing rabbits, and (ii) determine the apparent total tract digestibility and nutritive value of *A. domesticus* meal in a separate digestibility experiment. In Experiment 1, 50 growing New Zealand White rabbits were fed a control diet or a diet containing 3% *A. domesticus* meal from weaning (30–35 d) to slaughtering (88–93 days). In Experiment 2, 24 rabbits were used to determine the apparent total tract digestibility and nutritive value of *A. domesticus* meal using a diet containing 7.5% cricket meal. The 3% inclusion level did not significantly affect growth performance or feed conversion ratio. It increased duodenal villus width and jejunal and ileal villus height and was associated with lower histological inflammation scores in the small intestine (all *p* < 0.05). However, greater cecal goblet cell hyperplasia and hepatic fatty degeneration were also observed (*p* < 0.05). In Experiment 2, the diet containing 7.5% cricket meal increased dry matter, organic matter, and crude protein digestibility (all *p* < 0.05). *A. domesticus* meal showed high protein digestibility (86.2%) but relatively low gross energy digestibility (41.4%) and a moderate digestible energy value (2499 kcal/kg DM). Overall, *A. domesticus* meal did not impair growth performance and provided highly digestible protein, but further research is needed to clarify its energy utilization and intestinal and hepatic effects before its use as a partial protein replacement in rabbit diets.

## Simple Summary

As sustainable protein sources become increasingly important in livestock nutrition, house cricket (*Acheta domesticus*) meal has emerged as a promising alternative to conventional protein ingredients. However, its specific effects on rabbit growth performance and digestion require further evaluation. We conducted two separate experiments to assess *A. domesticus* meal as a partial replacement for soybean meal. In the feeding trial, a 3% inclusion level was not associated with significant differences in growth performance, feed intake, or feed conversion ratio. However, it was associated with changes in intestinal morphology, lower histological inflammation scores in the small intestine, increased cecal goblet cell hyperplasia, and greater hepatic fatty degeneration. In a separate digestibility trial, a 7.5% inclusion level increased the apparent digestibility of dietary dry matter, organic matter, and crude protein. The cricket meal itself showed high estimated apparent crude protein digestibility (86.2%), but relatively low estimated gross energy digestibility (41.4%). Overall, *A. domesticus* meal provides highly digestible protein without detectable adverse effects on rabbit growth, while its effects on intestinal and hepatic characteristics warrant further investigation.

## 1. Introduction

The increasing global demand for food and feed is placing substantial pressure on land, water, and other natural resources, highlighting the need for more resource-efficient and sustainable livestock production systems [1,2]. In this context, identifying alternative feed ingredients that can provide adequate nutrients while reducing dependence on conventional protein sources has become an important research priority [3,4,5]. Beyond their nutritional value, alternative feed ingredients may also influence animal physiology, digestive function, intestinal integrity, and overall health. Thus, evaluating their effects at both the productive and biological levels is essential for determining their suitability for sustainable animal production. Among the alternatives being investigated, insect-derived ingredients have attracted considerable interest as protein sources for animal nutrition [5,6]. Insects can efficiently convert feed substrates into biomass and generally require less land and water than conventional livestock production systems [7]. Their nutritional composition is also favorable, as many insect species contain substantial amounts of protein and lipids together with minerals, vitamins, and other bioactive components [8,9,10]. Consequently, insect-derived ingredients have been proposed not only as sources of dietary protein and energy but also as potential functional feed ingredients capable of influencing gastrointestinal and immune-related processes [11]. However, their nutritional and biological effects are not uniform and may vary according to insect species, developmental stage, processing conditions, and dietary inclusion level [7,12].

The house cricket (*Acheta domesticus*) is of particular interest because of its favorable nutritional characteristics and production efficiency [13]. Cricket meal contains high concentrations of crude protein and lipids, approximately 63–73% and 13–22% of dry matter, respectively, and provides a balanced amino acid profile [14]. The protein fraction of *A. domesticus* contains substantial amounts of essential amino acids, supporting its potential as a nutritionally valuable protein ingredient [15,16]. In addition, cricket protein has been reported to exhibit high digestibility, which may be related to the abundance and accessibility of muscle structural proteins [17]. House crickets also provide relevant amounts of minerals, including calcium, magnesium, iron, copper, manganese, and zinc, further contributing to their nutritional value [17]. Beyond their nutrient composition, house crickets have favorable feed conversion characteristics compared with conventional livestock species, supporting their potential contribution to more resource-efficient animal production systems [8,13]. However, the nutritive value of cricket meal cannot be inferred solely from its chemical composition, because nutrient utilization may be affected by structural components such as chitin, as well as by processing conditions and dietary inclusion level [13,18]. Chitin and chitosan may also exert biological effects within the gastrointestinal tract, including interactions with the intestinal microbial environment [19].

Although insect-derived ingredients have been investigated in several livestock species [20,21,22,23], the available evidence is dominated by black soldier fly (*Hermetia illucens*), yellow mealworm (*Tenebrio molitor*), silkworm (*Bombyx mori*), and housefly (*Musca domestica*) products [20,24,25,26]. Studies in poultry, swine, and aquaculture have shown that insect-derived meals and fats can partially replace conventional protein sources without necessarily compromising growth performance or nutrient utilization [27,28,29,30]. Importantly, some studies have also reported effects on intestinal morphology, gut microbiota, immune responses, and other indicators of gastrointestinal function [24,31,32,33]. These findings suggest that the biological relevance of insect-derived ingredients may extend beyond their contribution of protein and energy. However, such effects appear to be highly dependent on the insect species, ingredient composition, processing method, and dietary inclusion level [34,35], making species-specific investigations necessary.

The current EU framework therefore permits the use of processed animal protein derived from farmed insects in feed for aquaculture animals, poultry, and porcine animals, but does not include rabbits among the specified target species [36,37]. This regulatory context further underscores the need for species-specific evidence on the nutritional value, digestibility, and biological effects of insect-derived ingredients in rabbits [38].

Rabbits efficiently convert fibrous diets into high-quality, sustainable meat through specialized hindgut fermentation [39]. However, their unique digestive physiology is highly sensitive to dietary changes [40]. Therefore, the incorporation of alternative protein ingredients requires a comprehensive assessment of their effects on nutrient digestibility, gastrointestinal homeostasis, and overall productive performance [41,42]. This is particularly relevant when evaluating insect meal, because components of insect biomass, including chitin and other bioactive compounds, may interact with digestive and intestinal processes [43,44].

Based on the nutritional characteristics of *A. domesticus* meal and the available evidence on insect-derived ingredients, we hypothesized that partial replacement of soybean meal with *A. domesticus* meal would maintain growth performance and feed efficiency in growing rabbits, while inducing detectable changes in intestinal morphology and histopathological responses. We further hypothesized that *A. domesticus* meal would provide a highly digestible source of protein.

To test these hypotheses, the present study was designed as two independent experiments with distinct objectives. The first was a controlled feeding trial using a 3% inclusion level of *A. domesticus* meal as a partial replacement for soybean meal to evaluate its effects on growth performance, intestinal morphology, and intestinal and hepatic histopathological characteristics. The second was a separate in vivo digestibility trial using a diet containing 7.5% *A. domesticus* meal to determine its apparent total tract digestibility and nutritive value as an individual feed ingredient. By integrating growth performance, nutrient digestibility, intestinal morphology, and gastrointestinal and hepatic histopathology, this study aimed to characterize the effects and nutritional value of *A. domesticus* meal in growing rabbits and to provide evidence relevant to its use as an alternative protein ingredient in rabbit nutrition.

## 2. Materials and Methods

The present study comprised two separate experiments with distinct objectives, animal groups, inclusion levels, and experimental designs. Experiment 1 was a feeding trial designed to evaluate the effects of a 3% inclusion level of *A. domesticus* meal, incorporated as a partial replacement for soybean meal, on growth performance, intestinal morphology, and intestinal and hepatic histopathological characteristics. Experiment 2 was an independent digestibility trial conducted with a separate group of rabbits to determine the apparent total tract digestibility and nutritive value of *A. domesticus* meal. The 7.5% inclusion level used in Experiment 2 was selected as an appropriate level for the digestibility assessment using the substitution method and was not intended to represent a higher dose or a continuation of the 3% treatment used in Experiment 1. Accordingly, the two experiments were not designed to evaluate dose–response relationships or to determine an optimal dietary inclusion level.

The *A. domesticus* meal used in both experiments was obtained from the same commercial producer (Nutrinsect S.r.l., Montecassiano, Italy) and originated from the same production batch. Using the same ingredient batch ensured consistency in the cricket meal evaluated across the two experimental settings, while the separate experimental designs allowed its effects on growth performance and intestinal and hepatic characteristics to be assessed independently from its apparent total tract digestibility and nutritive value.

### 2.1. Experiment 1: Feeding Trial

#### 2.1.1. Animals, Housing, and Experimental Design

The feeding trial was conducted at the Faculty of Veterinary Medicine of the Agricultural University of Tirana. The experimental protocol was approved by the Ministry of Agriculture and Rural Development, National Authority of Veterinary and Plant Protection of Albania (Protocol No. 824/2021). All experimental procedures complied with national and international guidelines for the care and use of animals for scientific purposes. Animal health and welfare were monitored daily by the attending veterinarian throughout the experimental period, and all cases of morbidity and mortality were recorded.

An a priori power analysis was performed using G*Power (version 3.1) for the group × time interaction in body weight (BW). Assuming an effect size of f = 0.25, an α level of 0.05, 90% power, two dietary groups, two post-baseline repeated measurements, a correlation of 0.50 between repeated measures, and a nonsphericity correction of ε = 1, a minimum total sample size of 46 rabbits was estimated. To account for potential losses during the experimental period, 50 rabbits (25 per dietary treatment) were enrolled. Following mortality during the trial, 43 rabbits remained available for the growth performance analyses. A sensitivity analysis based on the final sample size and the same assumptions indicated that the study was able to detect a group × time interaction effect size of f = 0.253 or greater with 90% power.

Thus, a total of 50 weaned New Zealand White rabbits of both sexes were enrolled in the experiment. Rabbits from different litters were weaned at 30–35 days of age, depending on their date of birth, and housed individually in conventional wire cages (75 × 38 × 25 cm) until slaughter, 58 days after weaning (88–93 days of age). No environmental enrichment was provided. Animals were maintained under constant environmental conditions throughout the trial, with a temperature of 18–22 °C, 60% relative humidity, and a controlled photoperiod of 16 h light and 8 h dark.

At weaning, rabbits were allocated to one of two dietary treatments (*n* = 25 per treatment) using a body-weight-balanced allocation procedure. Rabbits were assigned alternately to the two dietary groups based on their initial body weight to ensure comparable baseline BW distributions. Sex was not considered during allocation, resulting in 10 females and 15 males in the CNT1 group and 12 females and 13 males in the ADM3 group. The two dietary treatments were: (i) a control diet (CNT1), consisting of a pelleted diet formulated with soybean meal as the principal protein source, and (ii) an *Acheta domesticus* meal diet (ADM3), in which 3% house cricket meal (*Acheta domesticus*) was incorporated as a replacement for soybean meal (Table 1).

The experimental diets were the same diets previously formulated, characterized, and used in the experimental work reported by Quattrone et al. [45]. Both diets were formulated to provide similar calculated digestible energy and crude protein concentrations and to meet the nutritional requirements of growing rabbits [42]. The 3% inclusion level was selected based on the available literature on insect-derived ingredients in rabbit diets, together with dietary formulation calculations, to provide a moderate partial replacement of soybean meal while limiting changes in the overall dietary composition. Specifically, 30 g/kg of soybean meal (80 g/kg in the control diet) was replaced with 30 g/kg of *A. domesticus* meal, corresponding to a 37.5% reduction in soybean meal inclusion. Thus, the 3% inclusion level was considered a literature-informed, formulation-based exploratory level for *A. domesticus* meal in growing rabbits, rather than an optimal or biologically established inclusion level, and was selected to provide a moderate contribution of cricket-derived components, including lipids and chitin, to the overall diet.

Throughout the experimental period, rabbits were offered pelleted diets in restricted amounts. The daily feed allowance was progressively increased from 90 to 120 g/rabbit/day according to age and production stage. Fresh drinking water was provided ad libitum.

#### 2.1.2. Chemical Analysis of Experimental Diets of the Feeding Trial

Representative samples of the experimental pelleted diets were collected after manufacture, vacuum-packed, transported to the laboratory, and finely ground prior to chemical analysis. The experimental diets were the same diets previously formulated and used in our earlier work [45], where their chemical composition was also characterized. Briefly, dry matter (DM), ash, crude protein (CP), ether extract (EE), and crude fiber (CF) were determined according to AOAC International procedures (Methods 934.01, 942.05, 984.13, 920.39, and 978.10, respectively). Crude protein was calculated as nitrogen × 6.25 following Kjeldahl analysis. Neutral detergent fiber (NDF), acid detergent fiber (ADF), and acid detergent lignin (ADL) were determined according to Van Soest et al. [46]. Chitin content was quantified using the sequential acid–alkali extraction procedure described by Psarianos et al. [47]. Nitrogen-free extract (NFE) was calculated by difference, and digestible energy (DE) was estimated according to the equation proposed by De Blas and Mateos [42]. The selected analyzed dietary characteristics considered most relevant to the interpretation of the present study are reported in Table 2. The complete chemical characterization of the *A. domesticus* meal used to formulate the ADM3 diet, including its ether extract and chitin concentrations, was previously reported in our earlier work [45].

#### 2.1.3. Mortality and Growth Performance

Body weight (BW) was recorded individually at three time points (T0–T2) during the experimental period. Measurements began at weaning (T0), were repeated 29 days later (T1), and were recorded again 29 days thereafter, immediately before slaughter (T2).

Body weight was measured in the morning before feed distribution using an electronic scale (Isola-Vignoli, Forlì, Italy) to minimize variation associated with gastrointestinal fill and feed intake.

Individual feed intake (FI) was recorded daily as the difference between the amount of feed offered and the corresponding feed refusals. No feed refusals were recorded during the experimental period; therefore, actual daily FI corresponded to the feed allowance.

Average daily gain (ADG) was calculated for each 29-day growth interval as the difference between BW at the beginning and end of the interval divided by the number of days, according to Equation (1).

Feed conversion ratio (FCR) was calculated for each corresponding growth interval as total feed intake divided by total BW gain, according to Equation (2), as previously described [48].

In addition, cumulative FCR was calculated for the entire experimental period (T0–T2) as the total feed intake accumulated over the 58-day period divided by the total BW gain from weaning to slaughter.

ADG (g/d) = (Final Body Weight (g) − Initial Body Weight (g))/Number of Days(1)

FCR = Total feed intake (g)/Total weight gain (g)(2)

Mortality was recorded throughout the experimental period. Rabbits that died during the study were excluded from the growth performance analyses. Of the 50 rabbits initially enrolled, 43 completed the experimental period and were included in the growth-performance analyses: 22 in the CNT1 group (9 females and 13 males) and 21 in the ADM3 group (10 females and 11 males).

#### 2.1.4. Intestinal Morphology and Intestinal and Hepatic Histopathology

At the end of the 58-day feeding period, rabbits that completed the feeding trial were slaughtered in a licensed commercial slaughterhouse in accordance with the national regulations governing the protection of animals at the time of killing. Immediately after slaughter, the gastrointestinal tract and liver were collected for histological and histopathological assessment from a subset of 24 randomly selected animals (12 per dietary treatment). A sensitivity power analysis was performed using G*Power for the intestinal morphometric analyses using the Wilcoxon–Mann–Whitney test. With 12 animals per dietary treatment, an α level of 0.05 and 80% power, the analysis indicated that the study was able to detect a standardized between-group effect size of d = 1.23 or greater. Thus, these analyses were powered to detect relatively large between-group differences.

Tissue samples were collected from the duodenum, jejunum, ileum, caecum, and liver. Samples were individually placed in sterile 15 mL tubes and fixed in 10% neutral buffered formalin for histological examination. Following fixation for 24 h, tissues were routinely processed, embedded in paraffin, sectioned at 3 μm, and mounted on Superfrost Plus slides (Histoline, Milan, Italy). Sections were deparaffinized, stained with hematoxylin and eosin (H&E), and mounted for observation under the optical microscope (Leica DM2500, Wetzlar, Germany).

H&E-stained sections of the duodenum, jejunum, and ileum were evaluated for intestinal morphometry. Five well-oriented villi from each intestinal segment were selected for each sampled animal, and villus height (VH), villus width (VW), and crypt depth (CD) were measured using an image-processing and analysis system (Leica Imaging Systems, Ltd., Cambridge, UK), as previously described [49].

VH was measured from the top of the villus to the top of the lamina propria [50]. CD was measured from the base upward to the region of transition between the crypt and villus [51]. VW was measured at the widest area of each villus, whereas the villus height-to-crypt depth ratio (VH/CD) was calculated as the ratio of VH to CD.

Histopathological evaluation included the following parameters: (i) inflammatory cell infiltration and enterocyte hyperplasia in the duodenum, jejunum, and ileum; (ii) goblet cell hyperplasia and epithelial hyperplasia in the cecum; and (iii) inflammatory cell infiltration and fatty degeneration in the liver. All histopathological alterations were graded using a 0–3 severity score, as described below.

For intestinal inflammatory cell infiltration, scores were assigned according to the number of inflammatory cells observed per microscopic field: 0 = 0–3 cells; 1 = 4–5 cells; 2 = 6–15 cells; and 3 = >15 cells [52].

Enterocyte and goblet cell hyperplasia was defined as an increase in the number of epithelial or goblet cells, respectively, relative to baseline cell numbers, occasionally associated with mitotic figures. Severity was graded according to the estimated percentage increase in cell number: 0 = <10%; 1 = 10–25%; 2 = >25–50%; and 3 = >50% [49].

The same 0–3 scoring criteria used for intestinal inflammatory cell infiltration were applied to liver samples to assess inflammatory cell infiltration within the hepatic parenchyma. In addition, hepatic fatty degeneration, characterized by microvesicular and/or macrovesicular steatosis, was graded according to the percentage of hepatocytes affected within the examined section: 0 = no affected hepatocytes; 1 = ≤33%; 2 = >33–66%; and 3 = >66% [53].

For each intestinal and liver sample, histological scoring was performed in three microscopic fields with 20× objective, a 10× eyepiece. Histological assessments were performed by an expert pathologist blinded to dietary treatment.

### 2.2. Experiment 2: Digestibility Trial

#### 2.2.1. Animals, Diets, and Experimental Design

A separate in vivo digestibility trial was conducted at the experimental rabbit facilities of the Instituto de Ciencia y Tecnología Animal (ICTA), Universitat Politècnica de València, Valencia, Spain. The experimental protocol was approved by the Ethical Committee of the Universitat Politècnica de València and authorized by the regional authority (Conselleria de Agricultura, Desarrollo Rural, Emergencia Climática y Transición Ecológica; approval code: 2025-VSC-PEA-0143).

This experiment was specifically designed to determine the apparent total tract digestibility (ATTD) of nutrients and the digestible nutrient content of *A. domesticus* meal using the substitution method described by Villamide et al. [54]. This method allows the nutritive value of an individual feed ingredient to be estimated indirectly by comparing the digestibility of a basal diet with that of a test diet in which a known proportion of the basal diet is replaced by the ingredient under evaluation. Accordingly, this experiment was designed to evaluate the nutritive value of *A. domesticus* meal as an individual feed ingredient and was not intended as a continuation of the 3% dietary treatment used in Experiment 1.

Twenty-four weaned rabbits (LP line, obtained from Universitat Politècnica de València, Valencia, Spain) of both sexes were individually housed in metabolic cages (29 × 50 × 32 cm) designed for quantitative fecal collection. No environmental enrichment was provided. Animals were maintained under controlled environmental conditions, with temperature maintained between 15 and 22 °C throughout the experimental period and automatic regulation of ventilation, and 62% relative humidity. A 12 h light:12 h dark photoperiod was maintained, with the light phase from 06:00 to 18:00. Animals had ad libitum access to feed and water throughout the experimental period.

A sensitivity power analysis was performed using G*Power for a repeated-measures design testing the between-group effect. With a total sample size of 24 rabbits (12 per dietary treatment), four repeated measurements, an α level of 0.05, 80% power, and an assumed correlation of 0.50 among repeated measurements, the study was able to detect an effect size of f = 0.47 or greater for the dietary treatment effect. The proposed sample size per treatment is consistent with the current recommendations of the European Group on Rabbit Nutrition for digestibility trials, which recommend a minimum of 10 animals per treatment to account for the expected variability in these traits [55].

From weaning, at 29 days of age, until the beginning of the experimental period at 42 days of age, rabbits were fed a commercial pelleted diet formulated for growing rabbits, with water available ad libitum. At 42 days of age, rabbits were weighed and randomly allocated to one of two dietary treatments (*n* = 12 per treatment) according to a randomized complete block design based on initial body weight.

Following a 7-day adaptation period (42–49 days of age), total fecal output was collected over four consecutive days (49–53 days of age) according to the standardized European reference method for in vivo digestibility trials in rabbits [56]. Individual BW was recorded at 42, 49, and 53 days of age. Feed intake was recorded daily throughout the experimental period. These measurements were used to calculate ADG, average daily feed intake (ADFI), and FCR. The corresponding performance data for the adaptation and collection periods are provided in Supplementary Table S1. Animal health and welfare were monitored daily throughout the experiment.

#### 2.2.2. Experimental Diets of the Digestibility Trial

Two experimental diets were formulated and manufactured at the ICTA pilot feed mill according to the nutritional recommendations for growing rabbits [42]. Following the substitution method described by Villamide et al. [54], *A. domesticus* meal was included at 7.5% of the test diet by replacing an equivalent proportion of the control basal diet, while the vitamin–mineral premix was maintained at the same inclusion level in both diets. The two diets were designated as CNT2 (control/basal diet) and ADM7.5 (test diet containing 7.5% *A. domesticus* meal), respectively. The ingredient composition and calculated nutrient composition of the experimental diets are presented in Table 3 and Table 4, respectively.

#### 2.2.3. Chemical Analysis

Representative samples of the experimental diets, *A. domesticus* meal, and fecal samples were analyzed in duplicate for dry matter (DM), ash, crude protein (CP), neutral detergent fiber (NDF), acid detergent fiber (ADF), acid detergent lignin (ADL), starch, and gross energy (GE). Dry matter, ash, and CP were determined according to AOAC International methods 934.01, 942.05, and 990.03, respectively. Fiber fractions were analyzed according to Mertens et al. [58] and Robertson and Van Soest [59], while starch was determined according to Batey [60]. Gross energy was measured using an adiabatic bomb calorimeter (Gallenkamp Autobomb, Loughborough, UK). The analyzed chemical composition of the CNT2 and ADM7.5 diets and *A. domesticus* meal is presented in Table 5 and Table 6, respectively.

#### 2.2.4. Digestibility Measurements and Calculations

Apparent total tract digestibility (ATTD) was determined according to the standardized European reference method for in vivo digestibility trials in rabbits [56]. During the four-day collection period, total fecal output was quantitatively collected daily, weighed, and stored at −20 °C until chemical analysis. Representative samples of the CNT2 and ADM7.5 diets were collected throughout the collection period to determine the actual nutrient composition of the diets offered and to calculate nutrient intake. Fecal samples were subsequently analyzed using the analytical procedures described above.

The ATTD coefficients of DM, organic matter (OM), crude protein (CP), and gross energy (GE) were calculated as the difference between nutrient intake and fecal nutrient excretion, expressed as a percentage of nutrient intake (Equation (3)):

(3) $$\mathrm{Apparent}\;\mathrm{digestibility}\;\mathrm{coefficient}\left(\%\right)=\left(\frac{\left(\mathrm{feed}\;\mathrm{intake}\times {\mathrm{nutrient}}_{\mathrm{diet}}\right)-(\mathrm{faecal}\;\mathrm{output}\times {\mathrm{nutrient}}_{\mathrm{faeces}})}{\mathrm{feed}\;\mathrm{intake}\times {\mathrm{nutrient}}_{\mathrm{diet}}}\right)\times 100$$

The apparent digestibility coefficients and digestible nutrient contents (and their respective standard errors) of the cricket meal were subsequently estimated using the substitution method described by Villamide et al. [54] (Equations (4)–(7)), where *DN_CF_* represents the digestible nutrient content of the cricket feed, *DN_BD_* the digestible nutrient content of the basal diet, *DN_CM_* the digestible nutrient content of the cricket meal, *DN_CD_* the digestible nutrient content of the control diet, and *DN_MM_* the digestible nutrient content of the mineral mix.

*DN_CF_* (g/kg) = *DN_BD_* (g/kg) × *Inclusion level of basal diet* + *DN_CM_* (g/kg) × *Inclusion level of cricket meal* (4)

*DN_CD_*(g/kg) = *DN_BD_*(g/kg) × *Inclusion level of basal diet* + *DN_MM_*(g/kg) × *Inclusion level of mineral mix* (5)

(6) $${\mathrm{DN}}_{\mathrm{CM}}\left(g/\mathrm{kg}\right)=\frac{{\mathrm{DN}}_{\mathrm{CF}}\left(g/\mathrm{kg}\right)-\left(\frac{{\mathrm{DN}}_{\mathrm{CD}}\left(g/\mathrm{kg}\right)}{0.99}\right)\times 0.915}{0.075}$$

(7) $$\mathrm{Digestibility}\;\mathrm{coefficient}\;\mathrm{of}\;\mathrm{nutrient}\left(\%\right)=\frac{{\mathrm{DN}}_{\mathrm{CM}}\left(g/\mathrm{kg}\right)}{\mathrm{Nutrient}\;\mathrm{in}\;\mathrm{cricket}\;\mathrm{meal}\left(g/\mathrm{kg}\right)}\times 100$$

### 2.3. Statistical Analysis

Data distributions and model assumptions were assessed using diagnostic plots and the Kolmogorov–Smirnov test. Extreme outliers were identified as observations falling more than three interquartile ranges below the first quartile (Q1 − 3 × IQR) or above the third quartile (Q3 + 3 × IQR). FCR values corresponding to intervals with zero or negative ADG were treated as missing because FCR is undefined when weight gain is zero and does not provide a biologically meaningful measure of feed conversion efficiency when weight gain is negative. No extreme outliers were identified for BW or ADG. Extreme values were identified for FCR but were retained because they were considered biologically plausible and were not attributable to apparent data-entry or measurement errors. However, the positively skewed distribution of FCR was accounted for in the subsequent GEE analysis.

Growth and productive performance data were analyzed using generalized estimating equations (GEE). Time (T1 and T2), dietary treatment (group), sex, and the group × time interaction were included as fixed effects, with an unstructured working correlation matrix specified to account for within-subject correlations over time. Body weight at weaning (T0) was included as a covariate in the BW model. BW and ADG were modeled using a normal distribution with an identity link, whereas FCR, which showed a positively skewed distribution, was modeled using a gamma distribution with a log link. In addition, cumulative FCR over the entire experimental period (T0–T2) was calculated for each rabbit as total feed intake over the 58-day period divided by total body weight gain over the same period. Because cumulative FCR represented a single value per animal, it was analyzed using a generalized linear model with a gamma distribution and log link, with dietary treatment included as a fixed effect. Results are presented as estimated marginal means ± standard error of the mean (SEM). Pairwise comparisons were adjusted using Sidak’s correction for multiple testing. Some individual BW measurements were unavailable because of technical issues during data recording, including illegible or implausible values that could not be reliably verified. Only valid measurements were retained for statistical analysis.

Differences in mortality between groups were assessed using Fisher’s exact test because of the small number of events.

For intestinal morphometry, the five measurements obtained for villus height, villus width, and crypt depth for each intestinal segment were averaged to obtain a single value per animal. Morphometric variables were then summarized at the group level as median and interquartile range (Q1–Q3) and compared between dietary treatments using the Mann–Whitney U test.

For histopathological variables, the scores obtained from the three microscopic fields evaluated for each animal and tissue were summarized using the median to obtain a single value per animal for statistical analysis. Histopathological scores were summarized as median and interquartile range and analyzed using proportional-odds ordinal logistic regression with a cumulative logit link function. Dietary treatment was included as a fixed effect, with the control diet (CNT) specified as the reference category. The proportional-odds assumption was evaluated using the test of parallel lines for outcomes with more than two observed score categories. Results from ordinal logistic regression are presented as odds ratios (ORs) with 95% confidence intervals (95% CIs). When complete or quasi-complete separation precluded reliable maximum-likelihood estimation of the ordinal logistic model, or when the proportional-odds assumption was violated, differences in score distributions between dietary treatments were assessed using exact tests (Fisher’s exact test for 2 × 2 tables or the Fisher–Freeman–Halton exact test for larger contingency tables).

Morphometric and histopathological variables represented distinct predefined biological outcomes and were analyzed separately. No global adjustment for multiple comparisons was applied across these heterogeneous outcomes; therefore, these analyses were considered exploratory, and borderline statistical findings were interpreted cautiously.

Statistical analyses for Experiment 1 were performed using IBM SPSS Statistics version 25 (IBM Corp., Armonk, NY, USA). Statistical significance was declared at *p* ≤ 0.05.

For Experiment 2, data were initially screened and explored prior to statistical analysis. Analyses were performed using SAS System Software version 9.4 (SAS OnDemand for Academics, Cary, NC, USA, 2023). Potential outliers were assessed using the UNIVARIATE procedure. The cage was considered the experimental unit, and treatment effects were evaluated using analysis of variance (ANOVA) with the GLM procedure, with dietary treatment included as the only fixed effect. Statistical significance was declared at *p* ≤ 0.05.

## 3. Results

### 3.1. Mortality and Growth Performance

During the experimental period, seven rabbits died (14.0%), with mortality rates of 12.0% (3/25) in the CNT1 group and 16.0% (4/25) in the ADM3 group, resulting in 22 and 21 rabbits completing the study, respectively; mortality did not differ between dietary treatments (Fisher’s exact test, *p* = 1.000).

No feed refusals were recorded in either dietary group during the experimental period; therefore, rabbits consumed the entire daily feed allowance. Accordingly, daily feed intake corresponded to the amount offered and was the same in both dietary groups, increasing from 90 to 110 and 120 g/rabbit/day over the experimental period.

Body weight (BW) was significantly affected by time (*p* < 0.001) and initial BW at weaning (*p* = 0.003), whereas no significant effects of dietary treatment (*p* = 0.311) or sex (*p* = 0.437) were observed. The group × time interaction was also not significant (*p* = 0.375; Table 7). Estimated marginal means increased from T1 to T2 in both dietary groups.

Average daily gain (ADG) was significantly affected by time (*p* < 0.001), decreasing from the T0–T1 to the T1–T2 growth interval. No significant effects of dietary treatment (*p* = 0.150), sex (*p* = 0.429), or the group × time interaction (*p* = 0.934) were observed (Table 7).

Feed conversion ratio (FCR) was significantly affected by time (*p* < 0.001), increasing from the T0–T1 to the T1–T2 growth interval. No significant effects of dietary treatment (*p* = 0.133), sex (*p* = 0.296), or the group × time interaction (*p* = 0.762) were observed (Table 7). No significant effect of dietary treatment was observed on cumulative FCR over the entire experimental period (ADM3: 4.54 ± 0.28; CNT1: 4.90 ± 0.24; *p* = 0.334).

### 3.2. Intestinal Morphometry and Histopathological Findings

Selected intestinal morphometric measurements differed between dietary treatments (Table 8). Compared with the CNT1 group, rabbits fed the ADM3 diet showed greater duodenal villus width (*p* < 0.001), greater jejunal villus height (*p* = 0.020) and crypt depth (*p* = 0.013), as well as greater ileal villus height (*p* = 0.002) and crypt depth (*p* = 0.020). The jejunal VH/CD ratio was lower in the ADM3 group than in the CNT1 group (*p* = 0.043). No significant differences were detected for duodenal villus height, crypt depth, or VH/CD ratio, jejunal villus width, or ileal villus width or VH/CD ratio (all *p* > 0.05).

**Figure 1:**
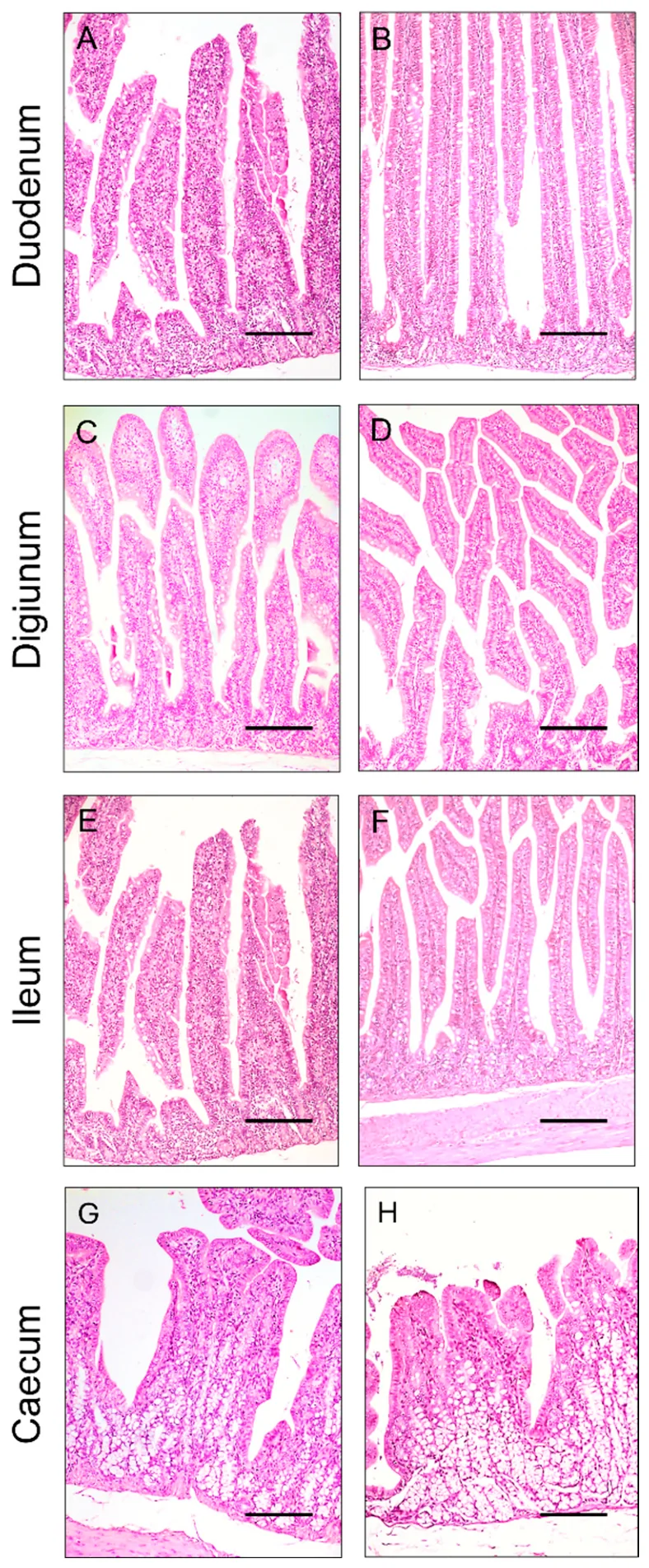
Representative histological appearance of the different intestinal segments evaluated in rabbits fed the control diet (CNT1; (**A**,**C**,**E**,**G**)) and the *Acheta domesticus* meal diet (ADM3; (**B**,**D**,**F**,**H**)). The images illustrate the histological parameters that significantly contributed to the differences between dietary groups in the odds ratio analysis. In particular, greater inflammatory cell infiltration is evident in the duodenum, jejunum, and ileum of rabbits in the CNT1 group (score 3 vs. 1) whereas goblet cell and epithelial hyperplasia is more pronounced in the ADM3 group (score 3 vs. 1). H&E staining; scale bars = 100 µm.

Histopathological evaluation revealed differences in intestinal mucosal morphology between dietary treatments (Figure 1). In the duodenum, jejunum, and ileum, inflammatory cell infiltration was more evident in CNT1 than in ADM3 rabbits. In the cecum, goblet cell hyperplasia was more prominent in ADM3-fed rabbits. These qualitative differences were observed across the intestinal segments and complemented the morphometric findings reported in Table 9.

**Figure 2:**
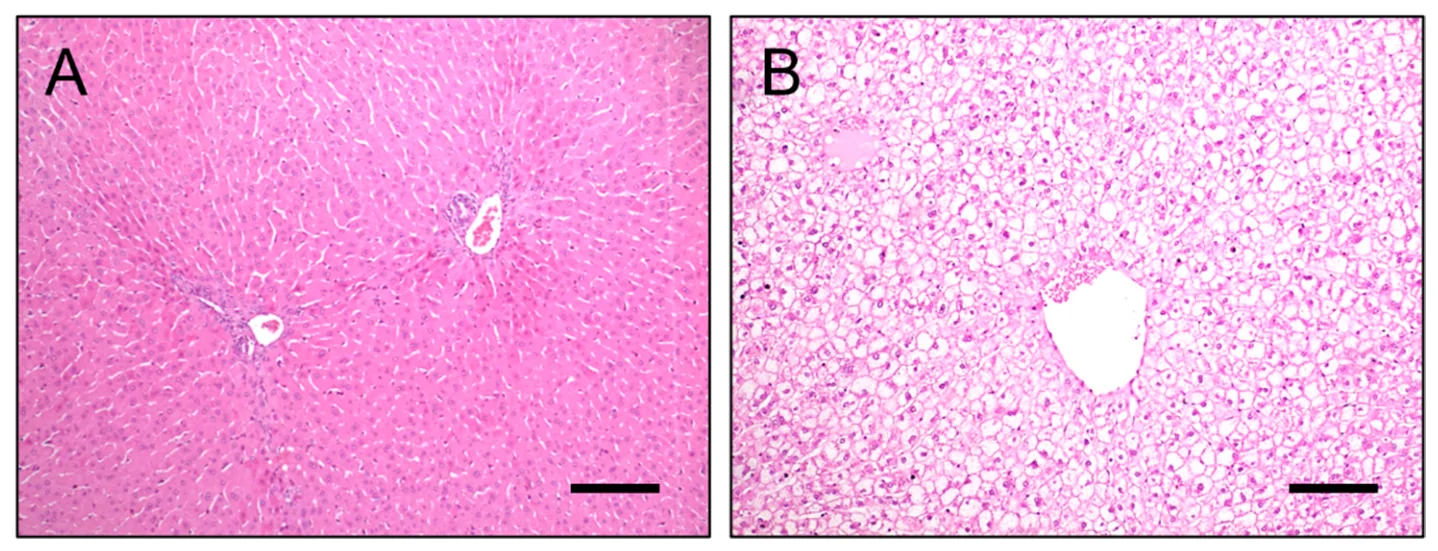
Representative liver histology from rabbits fed the control diet (**A**) or the *Acheta domesticus* meal diet (**B**). Fatty liver degeneration scores were 0 and 3, respectively, corresponding to 0% and >66% of the tissue being affected by the degenerative condition. H&E staining; scale bars = 100 µm.

Liver histology showed a marked difference in the extent of fatty degeneration between dietary groups (Figure 2). The CNT1 group had a fatty liver degeneration score of 0, indicating no affected tissue, whereas the ADM3 group had a score of 3, corresponding to involvement of more than 66% of the hepatic tissue.

**Figure 3:**
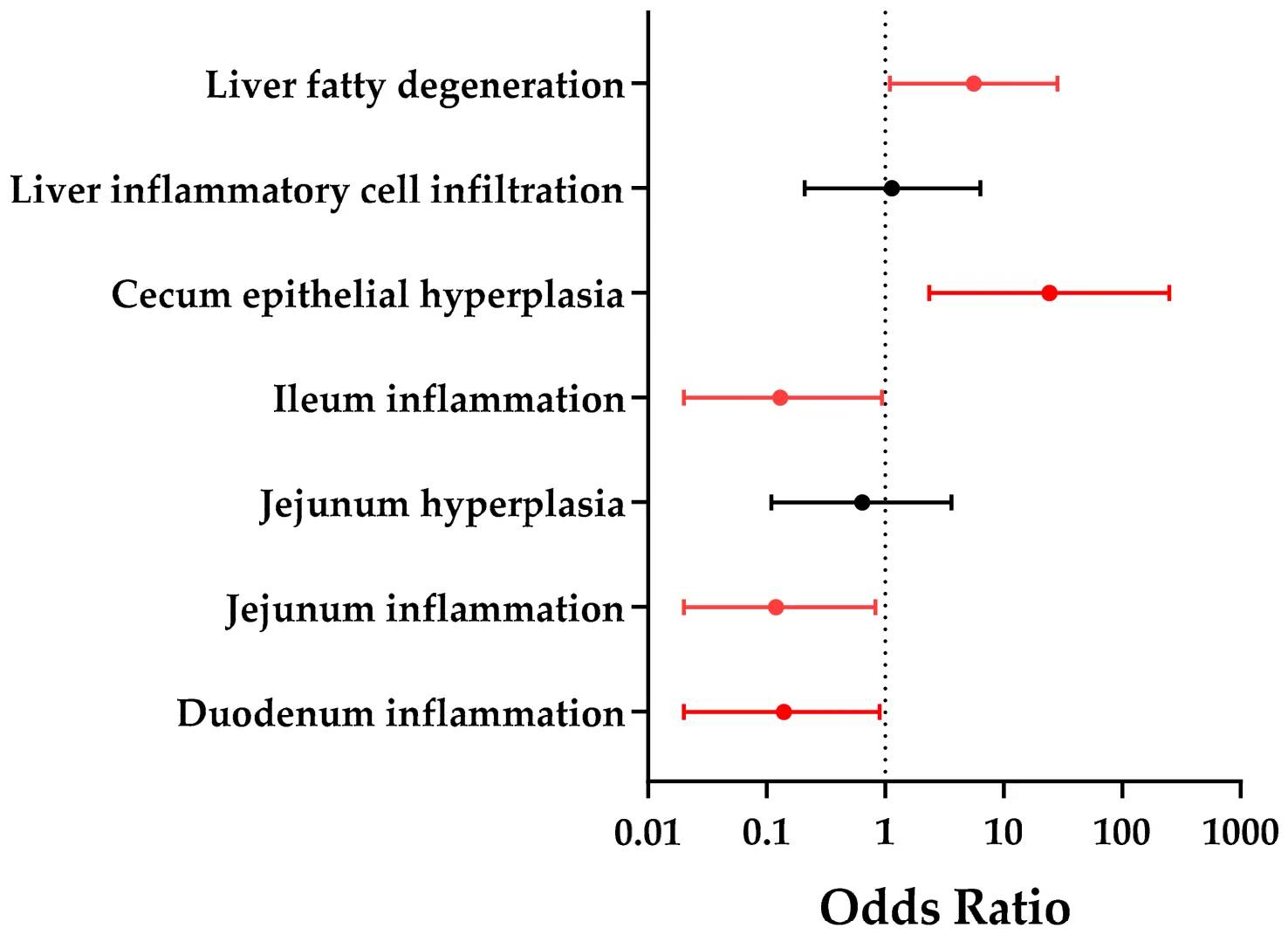
Odds ratios (OR) and 95% confidence intervals (95% CI) for histopathological scores in rabbits fed the *Acheta domesticus* meal diet (ADM3) compared with the control diet (CNT1). Odds ratios were estimated using proportional-odds ordinal logistic regression with CNT1 as the reference category. OR values < 1 indicate lower odds of higher histopathological scores in ADM3 compared with CNT1, whereas OR values > 1 indicate higher odds. Red symbols indicate statistically significant associations (*p* < 0.05). Duodenal and ileal hyperplasia are not shown because complete or quasi-complete separation precluded reliable OR estimation. Cecal goblet cell hyperplasia is not shown because the proportional-odds assumption was violated. The number of evaluable animals for each histopathological outcome and dietary group is reported in Table 9, with the corresponding score distributions provided in Supplementary Table S2.

Accordingly, the statistical analysis showed that several histopathological scores differed between dietary treatments (Table 9 and Figure 3; the corresponding raw score distributions reported in Table S2). Compared with the CNT1 group, rabbits fed the ADM3 diet had significantly lower odds of higher inflammatory scores in the duodenum (*p* = 0.039), jejunum (*p* = 0.032), and ileum (*p* = 0.043). Conversely, rabbits fed the ADM3 diet had higher odds of more severe cecal epithelial hyperplasia (*p* = 0.007) and liver fatty degeneration (*p* = 0.038). No significant differences in the odds of higher scores were observed for jejunal hyperplasia or liver inflammatory cell infiltration (both *p* > 0.05).

The proportional-odds assumption showed no evidence of violation for the applicable models (test of parallel lines, all *p* > 0.05), except for cecal goblet cell hyperplasia (*p* = 0.001). For this outcome, the proportional-odds OR was therefore not reported, and the Fisher–Freeman–Halton exact test indicated significantly higher scores in the ADM3 group (*p* = 0.002). For duodenal and ileal hyperplasia, complete or quasi-complete separation precluded reliable estimation of ORs; exact tests indicated significantly lower scores in the ADM3 group (*p* = 0.004 and *p* = 0.008, respectively).

### 3.3. Apparent Total Tract Digestibility and Nutritive Value of Acheta domesticus Meal

The apparent total tract digestibility coefficients (ATTDC) of dry matter (DM), organic matter (OM), crude protein (CP), and gross energy (GE) of the experimental diets, together with their digestible protein (DP) and digestible energy (DE) contents, are presented in Table 10.

Performance data recorded during Experiment 2 are provided in Supplementary Table S1. Initial and final BW did not differ significantly between dietary treatments (992 vs. 983 g at the beginning and 1505 vs. 1540 g at the end of the experimental period for CNT2 and ADM7.5, respectively; *p* = 0.891 and *p* = 0.593). Rabbits fed the ADM7.5 diet had a higher ADG than those fed the CNT2 diet (50.7 vs. 46.6 g/day; *p* = 0.001). In contrast, ADFI (128 vs. 123 g/day; *p* = 0.280) and FCR (2.54 vs. 2.70; *p* = 0.157) did not differ significantly between treatments (Table S1). Dry matter intake also did not differ significantly between dietary treatments (Table S1). However, rabbits fed the ADM7.5 diet had higher ATTDC values for DM (60.6 vs. 59.6%; *p* = 0.015), OM (59.0 vs. 57.7%; *p* = 0.003), and CP (73.7 vs. 70.7%; *p* < 0.001) than those fed the CNT2 diet. Accordingly, the DP content was higher in the ADM7.5 diet than in the CNT2 diet (136 vs. 104 g/kg DM; *p* < 0.001). In contrast, no significant differences between dietary treatments were observed for GE digestibility (*p* = 0.757) or DE content (*p* = 0.291). Mean GE digestibility was 58.9%, while mean DE content was 2716 kcal/kg DM.

The estimated ATTDC and digestible nutrient contents of *A. domesticus* meal, calculated using the substitution method described by Villamide et al. [48], are presented in Table 11. The estimated ATTDC of CP in *A. domesticus* meal was 86.2%, corresponding to a DP content of 530 g/kg DM. In contrast, the estimated ATTDC of GE was 41.4%, resulting in a DE content of 2499 kcal/kg DM.

## 4. Discussion

### 4.1. Growth Performance

In the present study, replacing part of the soybean meal with 3% *A. domesticus* meal was not associated with significant differences in growth performance in rabbits. Body weight (BW) and average daily gain (ADG) were not significantly affected by dietary treatment, and no significant group × time interactions were observed. BW increased over time in both groups, whereas ADG decreased during the later growth interval, consistent with the changes in growth rate expected as rabbits progressed through the fattening period. Additionally, daily feed intake did not differ between the two dietary groups, indicating that the inclusion of 3% *A. domesticus* meal did not negatively impact diet palatability or the animals’ feeding behavior, as already reported in previous studies using different insect-derived ingredients in rabbit diets [61,62].

Feed conversion ratio (FCR) changed significantly over time, with higher values during the later growth interval. However, neither the dietary treatment nor the group × time interaction was significant, and cumulative FCR over the entire experimental period did not differ between groups. The similar temporal pattern observed in both groups therefore suggests that the change in FCR was related to changes occurring over the growth period rather than to a treatment-specific response.

The absence of detrimental effects on growth performance is generally consistent with previous studies showing that insect-derived ingredients can be incorporated into rabbit diets without compromising productive performance. In particular, Volek et al. [61] reported that replacement of conventional protein sources with *A. domesticus* meal did not adversely affect body weight, daily weight gain, or feed efficiency in rabbits. However, the dietary strategies differed substantially between the two studies, as Volek et al. [61] investigated the complete replacement of conventional protein sources, whereas the present study evaluated a relatively low inclusion level of cricket meal as a partial substitute for soybean meal.

Similar findings have been reported with other insect-derived ingredients, although the effects have varied depending on the insect species, inclusion level, and dietary formulation. For example, Kowalska et al. [63] reported increased final BW and ADG in rabbits receiving diets containing 4% mealworm larvae or silkworm pupae meal, whereas Gugołek et al. [64] observed lower growth performance but improved FCR when soybean meal was partially or completely replaced with silkworm pupae meal. Similarly, Bui Phan Thu Hang et al. [65] reported favorable effects on growth performance and FCR when cricket meal replaced fish-meal protein at substantially higher replacement levels (25–100%) than those used in the present study. Other studies using insect-derived ingredients, including housefly larvae and mealworm or silkworm pupae meals, have likewise reported no detrimental effects on growth performance, feed efficiency, nutrient digestibility, gastrointestinal function, or carcass and meat quality traits [62,66,67,68]. Taken together, the available evidence suggests that the effects of insect meals on rabbit growth performance are not uniform across dietary formulations and cannot be directly extrapolated across different insect sources or replacement levels.

In the present study, the inclusion of 3% *A. domesticus* meal as a partial replacement for soybean meal also resulted in differences in diet composition, including ether extract, protein, and fatty acid profiles [45]. Accordingly, the observed responses should be interpreted as the overall effect of the dietary substitution rather than as effects of individual cricket-derived components.

Overall, the present results indicate that replacement of part of the soybean meal with 3% *A. domesticus* meal maintained growth performance under the conditions of this study. The relatively low inclusion level and partial replacement strategy may therefore represent a practical approach for incorporating cricket meal into rabbit diets without compromising productivity. Further studies evaluating multiple inclusion levels and nutritionally matched control diets are needed to determine whether responses vary with the proportion of soybean meal replaced and to better characterize the effects of *A. domesticus* meal on rabbit performance.

### 4.2. Intestinal Morphology

The intestinal findings indicate that replacing part of soybean meal with 3% *A. domesticus* meal was associated with distinct structural changes in the intestinal morphology. Rabbits fed ADM3 exhibited greater duodenal villus width and greater villus height in the jejunum and ileum, together with increased crypt depth in the jejunum and ileum. These changes may be relevant to intestinal function because villi provide the main epithelial surface for interaction with luminal nutrients. Greater villus height is generally associated with increased villus and mucosal surface area, while intestinal maturation involves coordinated changes in villus development and crypt architecture [69,70]. Thus, the greater villus height observed in the jejunum and ileum may indicate an expanded mucosal surface potentially available for nutrient absorption. This interpretation is consistent with previous findings in rabbits showing that dietary modulation of intestinal morphology may be accompanied by changes in functional indicators of nutrient utilization, such as digestive enzyme activity and ileal nutrient digestibility [71]. However, because absorptive capacity was not directly measured in the present study, the observed morphometric changes should be interpreted as structural adaptations potentially relevant to intestinal function rather than direct evidence of enhanced nutrient absorption.

The concurrent increase in crypt depth in the jejunum and ileum may reflect enhanced epithelial cell proliferation and mucosal renewal, as intestinal crypts constitute the principal site of epithelial cell production and replacement [72]. In the present study, the increase in crypt depth occurred together with greater villus height and lower inflammatory scores in the duodenum, jejunum, and ileum. Moreover, the absence of adverse effects on BW, ADG, and feed efficiency suggests that these structural changes were compatible with overall normal intestinal function.

The villus height-to-crypt depth (VH/CD) ratio did not differ significantly between dietary treatments in the duodenum or ileum, whereas it was lower in the jejunum of ADM3-fed rabbits. This reduction can be attributed to the proportionally greater increase in crypt depth relative to villus height in this intestinal segment. However, VH/CD values across all intestinal segments remained within ranges reported for rabbits [73,74], suggesting that the observed differences reflect alterations in villus–crypt architecture rather than mucosal impairment. Together with the increased jejunal villus height and reduced inflammatory scores, the lower VH/CD ratio may indicate adaptive mucosal remodeling rather than intestinal damage. However, the observed changes in villus height, crypt depth, and VH/CD ratio should be interpreted as alterations in villus–crypt architecture rather than as direct evidence of improved intestinal function or absorptive capacity.

Evidence specifically concerning intestinal responses to insect meal in rabbits remains scarce. Gasco et al. [35] reported that dietary replacement of soybean oil with fats derived from *Hermetia illucens* or *Tenebrio molitor* did not alter villus height, crypt depth, or their ratio, nor did it adversely affect intestinal or organ histopathology. The absence of detrimental intestinal effects is broadly consistent with our findings. However, their study involved purified insect fats rather than whole insect meal and is therefore not directly comparable with the present experiment. Moreover, the present study extends the available evidence by showing that a low inclusion level of whole *A. domesticus* meal (3%) was associated not merely with the absence of adverse morphological effects, but also with selective changes in villus and crypt architecture accompanied by lower intestinal inflammatory scores.

Another relevant response was the increase in cecal goblet cell hyperplasia in ADM3-fed rabbits. Goblet cells are specialized epithelial cells responsible for the synthesis and secretion of mucins, which form a protective mucus layer over the intestinal epithelium and contribute to epithelial barrier function, host–microbe interactions, and intestinal homeostasis [75]. The increased goblet cell hyperplasia may therefore represent an adaptive response to changes in the luminal environment associated with the cricket-containing diet. Similarly, epithelial hyperplasia observed in the cecal mucosa may reflect an adaptive and regenerative response to modifications in the intestinal microenvironment, potentially associated with microbial fermentation and increased epithelial turnover. Such a response could contribute to maintaining mucosal integrity and barrier function.

However, alterations in epithelial proliferation, goblet cell number and mucus production may also occur in response to intestinal challenge or inflammation [76]. In the present study, the interpretation of both epithelial and goblet cells hyperplasia as an adaptive response is supported by the simultaneous reduction in inflammatory scores in the small intestine and the absence of adverse effects on growth performance; nevertheless, direct measurements of mucin expression, mucus thickness, epithelial barrier integrity and microbial composition would be necessary to establish its functional significance.

A notable finding was also observed in the liver. Although inflammatory cell infiltration did not differ between dietary groups, ADM3-fed rabbits had higher odds of more severe hepatic fatty degeneration. The absence of increased hepatic inflammatory-cell infiltration suggests that the observed fatty degeneration was not accompanied by a detectable increase in this specific histological marker of hepatic inflammation. Consequently, the hepatic finding should be more appropriately interpreted as an alteration in hepatic lipid morphology rather than as evidence of generalized hepatic inflammation. Several mechanisms could potentially contribute to this alteration, including the specific lipid composition of the *A. domesticus* meal diet or other differences in dietary nutrient composition. Importantly, ADM3 had a higher analyzed ether extract content than CNT1 (26.4 vs. 20.8 g/kg), which should be considered when interpreting the increased hepatic fatty degeneration. Previous work using the same diets also reported differences in adiposity and fatty-acid composition [45]. However, as hepatic lipid metabolism and related metabolic markers were not assessed in the present study, a direct relationship between dietary ether extract and hepatic fatty degeneration cannot be established. A relevant comparison is provided by Gasco et al. [35], who reported multifocal to diffuse vacuolar degeneration of hepatocytes, together with periportal and/or interstitial lymphoplasmacytic inflammation, in rabbits fed diets containing *Hermetia illucens* or *Tenebrio molitor* fats. However, similar lesions were also observed in the control rabbits, and their severity did not differ among dietary treatments. The authors therefore considered the origin of these hepatic alterations uncertain and discussed their possible association with oxidative stress or other pathological conditions rather than attributing them directly to insect-derived fats [35]. In contrast, the present study identified a treatment-associated increase in the severity of hepatic fatty degeneration, although without a corresponding increase in inflammatory-cell infiltration. The difference between the two studies may partly reflect the nature of the insect ingredients evaluated: Gasco et al. [35] tested insect-derived fats as replacements for soybean oil, whereas the present study evaluated whole *A. domesticus* meal as a partial replacement for soybean meal, providing protein, lipids, chitin, and other insect-derived components.

A potential role of intestinal–hepatic interactions may be considered as a hypothesis for future investigation. The intestine and liver are physiologically connected through the portal circulation, and changes in intestinal microbial metabolism, epithelial barrier function, or luminal metabolites could potentially influence hepatic responses [77]. Insect-derived-ingredients may also modify the intestinal luminal environment through components such as chitin and other substrates [23]. Thus, it is conceivable that the ADM3 diet altered the intestinal luminal environment in a manner that affected hepatic metabolism. However, this interpretation remains hypothetical, as the present study did not assess microbiota composition, intestinal pH, short-chain fatty acids, intestinal permeability, or other gut-derived metabolites. Future studies should therefore combine intestinal morphometry with direct functional measurements, including epithelial barrier markers, mucin expression, digestive enzyme activity, microbiota composition, and relevant microbial metabolites. Assessment of serum biochemical indicators together with hepatic lipid composition and markers of oxidative stress, inflammation, and cellular injury would also help determine the biological significance of the hepatic fatty degeneration observed in ADM3-fed rabbits. Such studies would be particularly useful for distinguishing effects attributable to whole *A. domesticus* meal from those associated with the accompanying changes in dietary lipid and other nutrient fractions.

Overall, given the limited evidence available in rabbits and the relatively small sample size used for the histological assessment, these findings should be considered exploratory. In particular, the sample size of 12 animals per treatment limits the statistical power to detect small or moderate effects. Therefore, the observed changes in intestinal morphology and histopathology should be interpreted cautiously as exploratory observations rather than as definitive evidence of improved intestinal function or absorptive capacity. Further studies involving larger populations and direct functional measurements are warranted to determine whether these morphological changes translate into measurable changes in intestinal function.

### 4.3. Digestibility Trial

A major finding of the present study was the improvement in the apparent total tract digestibility coefficients (ATTDC) of dry matter (DM), organic matter (OM), and, particularly, crude protein (CP) in rabbits fed the ADM7.5 diet. The higher CP digestibility indicates an efficient utilization of cricket-derived proteins by the rabbit digestive system. Furthermore, the calculated apparent CP digestibility coefficient of *A. domesticus* meal itself reached 86.2%, demonstrating a very high protein availability [78,79]. These results are particularly relevant considering the limited information currently available regarding the digestibility of cricket meal in rabbits. The observed CP digestibility is indeed comparable to values reported for highly digestible conventional animal protein sources, such as high-quality fish meal, which typically exhibits CP digestibility coefficients close to 85% in rabbits [80,81]. Previous studies evaluating insect-derived ingredients in rabbits reported good protein digestibility and amino acid utilization when *Hermetia illucens* and *Tenebrio molitor* meals were included as alternative protein sources [34,82]. The high protein digestibility observed in the present study may be related to the favorable amino acid profile and high protein concentration of cricket meal, which contains substantial levels of essential amino acids comparable to those of conventional animal proteins [83,84]. The ability of rabbits to efficiently utilize insect-derived proteins is also supported by findings obtained in other monogastric species. In poultry and aquaculture, insect meals have consistently shown high protein digestibility and satisfactory nutrient utilization due to their balanced amino acid composition and high protein quality [11,20,85,86].

The increase in DM and OM digestibility observed in the ADM7.5 diet is also noteworthy. These findings indicate that inclusion of *A. domesticus* meal did not impair, and may have modestly improved, the overall apparent utilization of dietary nutrients. However, the magnitude of the response was greater for CP than for DM and OM, while GE digestibility remained unchanged between diets. This pattern suggests that the main nutritional benefit of incorporating cricket meal in the present study was related to protein utilization rather than a generalized improvement in energy utilization. The higher CP digestibility may reflect the high digestibility and accessibility of insect-derived protein, whereas the lack of improvement in GE digestibility indicates that the additional protein supplied by cricket meal did not translate into greater overall energy availability [17,87].

In contrast to protein digestibility, the apparent GE digestibility of cricket meal was relatively low (41.4%), resulting in a moderate DE value (2499 kcal/kg DM). Moreover, no differences in GE digestibility were detected between the experimental diets despite the higher digestible protein content of the cricket diet. Similar findings have been reported in rabbits fed insect-derived ingredients, where protein digestibility improved while energy digestibility remained unchanged or decreased [34,35]. The relatively low GE digestibility observed in the present study is likely associated with the chitin fraction contained in the insect exoskeleton. Chitin is a nitrogen-containing structural polysaccharide that is considerably more resistant to endogenous enzymatic digestion than protein and may contribute to the less digestible fraction of insect meals [23]. Although rabbits possess a highly specialized hindgut fermentation system capable of efficiently utilizing fibrous substrates, the degradation of chitin might remain limited [20,35]. Consequently, part of the gross energy contained in cricket meal may not be fully available to the animal. The effect of chitin on nutrient utilization has also been discussed in studies involving other insect species. Gasco et al. [35] observed that increasing dietary inclusion levels of insect fats in rabbits negatively affected the digestibility of dry matter, ether extract, and gross energy. Similarly, Dalle Zotte et al. [34] suggested that the presence of insect structural components may influence nutrient digestibility and energy utilization. The extent of these effects may vary depending on insect species, developmental stage, and processing methods, all of which can influence chitin concentration and nutrient accessibility [23,88].

From a nutritional perspective, the highly digestible protein content of cricket meal (530 g/kg DM) highlights its potential as an alternative protein source capable of partially replacing conventional ingredients such as soybean meal or fish meal in rabbit diets. However, its relatively lower digestible energy value indicates that dietary formulations including cricket meal should be carefully balanced to ensure adequate energy supply, particularly when high inclusion levels are considered.

Several limitations should be considered when interpreting the present findings. The relatively small sample sizes of the histological and digestibility assessments limit the precision and generalizability of these findings and warrant confirmation in larger populations. The two experiments had different objectives, experimental designs, and dietary inclusion levels (3% and 7.5%, respectively); therefore, their results should be interpreted within the context of their respective experimental approaches rather than as a direct comparison of inclusion levels. Although the feeding trial provided evidence of changes in intestinal morphology and histopathological characteristics, the functional significance of these findings was not directly established and warrants further investigation. Furthermore, the digestibility experiment was designed to determine the apparent total tract digestibility and nutritive value of *A. domesticus* meal and was not intended to evaluate its long-term physiological effects. Future studies should therefore investigate different inclusion levels over longer feeding periods and combine productive and digestibility measurements with functional assessments of intestinal barrier integrity, microbiota, mucin-related responses, and hepatic lipid and metabolic status. Such studies would help clarify the functional significance of the intestinal changes observed in the present study, provide further insight into the biological relevance of the hepatic fatty degeneration associated with *A. domesticus* meal inclusion, and help identify an appropriate inclusion level for its long-term use in rabbit production.

## 5. Conclusions

The present study provides new evidence on the nutritional and biological effects of house cricket (*Acheta domesticus*) meal in fattening rabbits. At an inclusion level of 3% as a partial replacement for soybean meal, *A. domesticus* meal did not adversely affect body weight, average daily gain, or feed conversion ratio under the conditions of the present experiment. Its inclusion was associated with segment-specific changes in intestinal villus–crypt architecture and with lower histopathological inflammatory scores in the small intestine. However, increased cecal goblet cell hyperplasia and greater hepatic fatty degeneration were also observed, while hepatic inflammatory-cell infiltration did not differ between dietary treatments. Although these histological findings were obtained from a relatively small number of animals and should therefore be interpreted cautiously, they provide interesting indications of potential intestinal and hepatic responses to *A. domesticus* meal that warrant further investigation in larger studies.

The separate digestibility trial demonstrated high apparent crude protein digestibility of *A. domesticus* meal (86.2%), corresponding to 530 g digestible protein/kg DM, and increased apparent digestibility of dry matter, organic matter, and crude protein in the diet. However, its relatively low gross energy digestibility (41.4%) indicates that its contribution to dietary energy should be considered during feed formulation.

Overall, under the experimental conditions of the present study, *A. domesticus* meal at the 3% inclusion level evaluated as a partial replacement for soybean meal showed potential as a highly digestible protein source, with no evidence of adverse effects on growth performance. However, its nutritional value and suitability should be considered in relation to its effects on productive performance, nutrient utilization, intestinal morphology and histopathology, and hepatic tissue responses. Further studies involving larger populations, different inclusion levels, and longer feeding periods are warranted to confirm these findings and determine their biological significance and long-term implications.

## Figures and Tables

**Table 1:** Ingredient composition of the control (CNT1) and *Acheta domesticus* meal (ADM3) diets used in the feeding trial.

Ingredients (g/kg)	CNT1	ADM3
House cricket (*Acheta domesticus*) meal	-	30.0
Corn grain	200.0	200.0
Barley	180.0	180.0
Soybean meal	80.0	50.0
Wheat bran	150.0	150.0
Alfalfa hay	340.0	340.0
Sunflower oil	10.0	10.0
Cane molasses	20.0	20.0
Calcium carbonate	9.0	9.0
Vitamin–mineral premix ^1^	5.0	5.0
Lysine	2.3	2.3
Methionine + Cysteine	1.5	1.5
L-Threonine	0.7	0.7
Sodium chloride	1.5	1.5

CNT1: control diet; ADM3: diet containing 3% *Acheta domesticus* meal as a partial replacement for soybean meal. ^1^ Vitamin–mineral premix per kg diet: vitamin A 11,000 IU; vitamin D3 2000 IU; vitamin B1 2.5 mg; vitamin B2 4 mg; vitamin B6 1.25 mg; vitamin B12 0.01 mg; alpha-tocopherol acetate 50 mg; biotin 0.06 mg; vitamin K 2.5 mg; niacin 15 mg; folic acid 0.30 mg; D-pantothenic acid 10 mg; choline 600 mg; Mn 60 mg; Fe 50 mg; Zn 15 mg; I 0.5 mg; Co 0.5 mg.

**Table 2:** Selected analyzed chemical composition of the control (CNT1) and *Acheta domesticus* meal (ADM3) diets used in the feeding trial.

Component (g/kg)	CNT1	ADM3
Dry matter (DM)	933.2	925.1
Ether extract (EE)	20.8	26.4
Crude protein (CP)	171.2	174.6
Crude Fiber (CF)	103.5	102.7
Ash	63.2	60.3
Chitin	-	1.9
Digestible Energy (DE) (kcal/kg) ^1^	2268.68	2270.32

CNT1: control diet; ADM3: diet containing 3% *Acheta domesticus* meal as a partial replacement for soybean meal. ^1^ Digestible Energy-DE (kcal/kg) was estimated according to De Blas and Mateos [42]. Note: The selected parameters are reported to facilitate interpretation of the present study, particularly with respect to dietary protein, lipid, chitin, and energy supply. The complete chemical characterization of the experimental diets was previously reported [45].

**Table 3:** Ingredient composition of the control and *Acheta domesticus* meal (ADM) diets used in the digestibility trial.

Ingredients (g/kg)	CNT2	ADM7.5
Barley grain	197	182
Wheat bran	200	185
Soybean meal	60.0	55.5
Cricket meal	-	75.0
Alfalfa hay	250	231
Beet pulp	100	92.4
Soybean hulls	40	37.0
Cereal straw	110	102
Soybean oil	30.0	27.7
L-lysine HCl	1.00	0.92
DL-methionine	1.00	0.92
L-Threonine	1.00	0.92
Sodium bicarbonate	3.50	3.50
Sodium chloride	2.00	2.00
Dicalcium phosphate	0.50	0.50
Vitamin-trace elements premix ^1^	4.00	4.00

CNT2: control/basal diet; ADM7.5: diet containing 7.5% *Acheta domesticus* meal used in the digestibility trial. ^1^ Composition of mineral-vitamin premix per kg: Vitamin A: IU/kg 2,500,000, Vitamin D3: 375,000 IU, Vitamin E: 5000 mg, Vitamin B12: 3.0 mg, Vitamin B6: 250 mg, Vitamin B1: 250 mg, Vitamin B2: 1.0 g, Niacin: 5.0 g, D-pantothenic acid: 2.5 g, Choline chloride: 2.5 g, Betaine (betaine hydrochloride): 11.52 g, Iron (iron carbonate): 12.5 g, Manganese (manganese oxide): 12.5 g, Selenium (sodium selenite): 25 mg, Zinc (zinc oxide): 15.0 g, Copper (copper sulphate, pentahydrate): 2.5 g, Iodine (potassium iodide): 250 mg, Cobalt (cobalt carbonate): 75 mg. Note: The ADM7.5 test diet was formulated by replacing an equivalent proportion of the basal diet with 75 g/kg *A. domesticus* meal, while maintaining the vitamin–trace element premix at the same inclusion level in both diets.

**Table 4:** Calculated chemical composition of the experimental diets used in the digestibility trial (according to FEDNA, 2019 [57]).

Calculated Nutrients (g/kg)	CNT2	ADM7.5
Digestible energy (kcal/kg)	2365	2449
Crude protein	141	174
Neutral detergent fibre	369	364
Acid detergent fibre	220	211
Acid detergent lignin	44.9	44.0
Starch	154	143
Ether extract	51.9	67
Calcium	6.42	6.13
Phosphorus	4.11	4.24
Sodium	3.90	4.04
Chlorine	4.49	4.55

CNT2: control/basal diet; ADM7.5: diet containing 7.5% *Acheta domesticus* meal.

**Table 5:** Analyzed chemical composition of the experimental diets used in the digestibility trial.

Analyzed Nutrient (g/kg Dry Matter)	CNT2	ADM7.5
Dry matter	892	892
Ashes	69.9	68.5
Crude protein	147	184
Neutral detergent fibre	377	348
Acid detergent fibre	203	178
Acid detergent lignin	12.2	10.1
Starch	186	170
Gross energy (kcal/kg dry matter)	4625	4602

**Table 6:** Analyzed nutrient composition of *Acheta domesticus* meal used in the digestibility trial.

Analyzed Nutrient (g/kg Dry Matter)	*Acheta domesticus* Meal
Dry matter	930
Ashes	64.5
Crude protein	615
Neutral detergent fibre	134
Acid detergent fibre	54.9
Acid detergent lignin	0.10
Starch	16.4
Gross energy (kcal/kg dry matter)	6031

**Table 7:** Body weight (BW), average daily gain (ADG), and feed conversion ratio (FCR) in rabbits from the feeding trial (Experiment 1), presented as observed or estimated marginal means ± SEM with the number of valid observations (*n*).

Variable	Group	Time			Significance				
		T0 *	T1	T2	BW at Weaning	Sex	Group	Time	Group × Time
BW (kg)	CNT1	0.99 ± 0.07 (*n* = 22)	2.01 ± 0.05 (*n* = 22)	2.41 ± 0.05 (*n* = 22)	0.003	0.437	0.311	<0.001	0.375
	ADM3	0.91 ± 0.09 (*n* = 21)	2.04 ± 0.05 (*n* = 21)	2.51 ± 0.06 (*n* = 21)					
ADG (g/day)	CNT1	–	35.36 ± 2.08 (*n* = 22)	13.75 ± 1.53 (*n* = 22)	-	0.429	0.150	<0.001	0.934
	ADM3	–	38.32 ± 2.61 (*n* = 21)	16.32 ± 2.26 (*n* = 21)					
FCR	CNT1	–	3.55 ± 0.34 (*n* = 22)	9.23 ± 0.63 (*n* = 21)	-	0.296	0.133	<0.001	0.762
	ADM3	–	3.14 ± 0.20 (*n* = 21)	7.64 ± 1.32 (*n* = 18)					

CNT1, control diet; ADM3, diet containing 3% *Acheta domesticus* meal as a partial replacement for soybean meal; BW, body weight; ADG, average daily gain; FCR, feed conversion ratio. * T0 values represent raw means (±SEM) of BW at weaning and were not estimated from the GEE model. BW at weaning was included as a covariate in the model and was fixed at its overall mean value (0.952 kg) for the estimation of marginal means at subsequent time points.

**Table 8:** Morphometric measurements of the intestinal mucosa in the duodenum, jejunum, and ileum of rabbits fed the control diet (CNT1) or the *Acheta domesticus* meal diet (ADM3). Values are presented as median (interquartile range, Q1–Q3).

Intestinal Segment	Variable	Group		*p* Value
		CNT1	ADM3	
Duodenum	Villus height (µm)	1006.66 (965.68–1139.93)	966.86 (824.67–1096.42)	0.251
	Villus width (µm)	99.37 (89.15–106.20)	118.65 (107.54–124.47)	<0.001
	Crypt depth (µm)	131.02 (111.33–137.27)	140.15 (114.78–173.87)	0.152
	VH/CD	8.39 (7.54–9.08)	7.89 (4.77–8.49)	0.152
Jejunum	Villus height (µm)	686.61 (655.05–770.07)	959.63 (721.78–1109.42)	0.020
	Villus width (µm)	88.40 (74.73–118.54)	108.39 (101.91–126.75)	0.197
	Crypt depth (µm)	81.31 (67.58–92.87)	143.98 (111.04–148.26)	0.013
	VH/CD	8.52 (8.14–9.21)	6.62 (6.13–7.85)	0.043
Ileum	Villus height (µm)	631.27 (539.45–725.28)	941.59 (702.27–1221.74)	0.002
	Villus width (µm)	90.55 (83.64–112.81)	94.25 (81.92–99.49)	0.941
	Crypt depth (µm)	86.73 (76.72–116.23)	116.95 (104.10–161.62)	0.020
	VH/CD	6.79 (6.35–7.28)	7.25 (5.83–9.30)	0.456

The number of evaluable animals varied among intestinal segments because of tissue availability and section quality: CNT1 *n* = 10 and ADM3 *n* = 11 for the duodenum and jejunum, and CNT1 *n* = 9 and ADM3 *n* = 11 for the ileum. Differences between dietary treatments were assessed using the Mann–Whitney U test.

**Table 9:** Histopathological scores in rabbits fed the control diet (CNT1) or the *Acheta domesticus* meal diet (ADM3). Data are presented as median (Q1–Q3). Odds ratios (ORs) and 95% confidence intervals (CIs) were estimated by proportional-odds ordinal logistic regression, with CNT1 as the reference group; OR > 1 indicates higher and OR < 1 lower odds of a higher histopathological score in ADM3. Exact tests were used when ordinal regression was not applicable.

Histological Parameter	Group		OR (95% CI)	*p*
	CNT1	ADM3		
Duodenum inflammation	1 (0–1)	0 (0–1)	0.14 (0.02–0.90)	0.039
Duodenum hyperplasia	1 (0–1)	0 (0–0)	-	0.004 ^a^
Jejunum inflammation	1 (1–1)	0 (0–1)	0.12 (0.02–0.83)	0.032
Jejunum hyperplasia	0 (0–1)	0 (0–1)	0.64 (0.11–3.63)	0.613
Ileum inflammation	1 (1–1)	0 (0–1)	0.13 (0.02–0.94)	0.043
Ileum hyperplasia	1 (0–1)	0 (0–0)	-	0.008 ^b^
Cecum goblet cell hyperplasia	1 (0–1)	2 (1–2)	-	0.002 ^b^
Cecum epithelial hyperplasia	1 (0–1)	2 (1–2)	24.35 (2.37–250.45)	0.007
Liver inflammatory cell infiltration	0 (0–1)	0 (0–1)	1.00 (0.18–5.67)	1.000
Liver fatty degeneration	1 (0–2)	2 (1–3)	5.60 (1.10–28.53)	0.038

^a^ Fisher’s exact test; ^b^ Fisher–Freeman–Halton exact test. The number of evaluable animals was CNT1 *n* = 10 and ADM3 *n* = 11 for the duodenum and jejunum, CNT1 *n* = 9 and ADM3 *n* = 11 for the ileum, and CNT1 *n* = 11 and ADM3 *n* = 12 for the cecum. For the liver, CNT1 *n* = 11 for both outcomes, whereas ADM3 n = 11 for inflammatory cell infiltration and *n* = 12 for fatty degeneration. The number of evaluable animals varied among tissues because of tissue availability and section quality. Raw frequencies and percentages of animals assigned to each histopathological score category are provided in Table S2.

**Table 10:** Apparent total tract digestibility coefficients (ATTDC) of dry matter (DM), organic matter (OM), crude protein (CP), and gross energy (GE), together with digestible protein (DP) and digestible energy (DE) contents of the experimental diets used in the digestibility trial. Each dietary treatment included 12 rabbits.

Treatment	DMI (g DM/day) ^2^	DM Dig (%)	OM Dig (%)	CP Dig (%)	GE Dig (%)	DP (g/kg of DM)	DE (kcal/kg of DM)
CNT2	113.3	59.6	57.7	70.7	58.9	104	2725
ADM7.5	112.8	60.6	59.0	73.7	58.8	136	2706
SEM ^1^	2.64	0.28	0.29	0.51	0.28	0.8	13.0
*p*-Value	0.909	0.015	0.003	<0.001	0.757	<0.001	0.291

^1^ SEM = Standard error of the mean; ^2^ DMI = DM intake.

**Table 11:** Estimated apparent total tract digestibility coefficients (ATTDC) of crude protein (CP) and gross energy (GE), and digestible protein (DP) and digestible energy (DE) contents of *Acheta domesticus* meal. Values are presented as estimated value ± standard error (SE).

	CP Dig (%)	GE Dig (%)	DP (g/kg of DM)	DE (kcal/kg of DM)
*Acheta domesticus* meal	86.2 ± 1.6	41.4 ± 1.4	530 ± 6	2499 ± 72

## Data Availability

The data supporting the findings of this study are provided in the article and its Supplementary Materials. The raw data are available from the corresponding author upon reasonable request.

## Supplementary Materials

The following supporting information can be downloaded at: https://www.mdpi.com/article/10.3390/biology15201898/s1, Table S1: Body weight and growth performance of rabbits from 42 to 53 days of age in Experiment 2; Table S2: Distribution of histopathological scores in rabbits fed the control diet (CNT1) or the *Acheta domesticus* meal diet (ADM3). Data are presented as the number and percentage of animals within each dietary group assigned to each score category, *n* (%). The number of evaluable animals varied among tissues and histopathological outcomes because of missing observations.
