# Supplementary material for: House Cricket (Acheta domesticus) Meal as an Alternative Protein Source for Fattening Rabbits: Effects on Growth Performance, Nutrient Digestibility, Intestinal Morphology, and Histopathology
Source: Biology (Basel). 2026 Oct 21;15(20):1898. doi: 10.3390/biology15201898 (PMC13701613; doi:10.3390/biology15201898)
Supplement: Supplementary file 1 [file biology-15-01898-s001.zip › biology-4548946-supplementary.pdf]

Supplementary Materials

Table S1. Body weight and growth performance of rabbits from 42 to 53 days of age in Experiment 2.

Group	BW at 42 d (g)	BW at 53 d (g)	ADG (g/day)	ADFI (g/day)	FCR
CNT2	992	1505	46.6	123	2.70
ADM7.5	983	1540	50.7	128	2.54
SEM[1]	46.6	46.1	0.78	3.1	0.075
P Value	0.891	0.593	0.001	0.280	0.157

[1]SEM = Standard error of the mean. *n* = 24 (12 rabbits/group). BW, body weight; ADG, average daily gain; ADFI, average daily feed intake; FCR, feed conversion ratio; CNT2, control diet; ADM7.5, diet containing 7.5% *A. domesticus* meal.

Table S2. Distribution of histopathological scores in rabbits fed the control diet (CNT1) or the *Acheta domesticus* meal diet (ADM3). Data are presented as the number and percentage of animals within each dietary group assigned to each score category, n (%). The number of evaluable animals varied among tissues and histopathological outcomes because of missing observations.

Histopathological parameter	Score	CNT1, n (%)	ADM3, n (%)
Duodenum inflammation	0	3 (30.0)	8 (72.7)
	1	5 (50.0)	3 (27.3)
	2	2 (20.0)	0 (0.0)
Duodenum hyperplasia	0	4 (40.0)	11 (100.0)
	1	6 (60.0)	0 (0.0)
Jejunum inflammation	0	2 (20.0)	7 (63.6)
	1	6 (60.0)	4 (36.4)
	2	2 (20.0)	0 (0.0)
Jejunum hyperplasia	0	6 (60.0)	7 (63.6)
	1	2 (20.0)	4 (36.4)
	2	2 (20.0)	0 (0.0)
Ileum inflammation	0	1 (11.1)	7 (63.6)
	1	7 (77.8)	3 (27.3)
	2	0 (0.0)	1 (9.1)
	3	1 (11.1)	0 (0.0)
Ileum hyperplasia	0	4 (44.4)	11 (100.0)
	1	4 (44.4)	0 (0.0)
	2	1 (11.1)	0 (0.0)
Cecum goblet cell hyperplasia	0	3 (27.3)	1 (8.3)
	1	8 (72.7)	3 (25.0)
	2	0 (0.0)	8 (66.7)
Cecum epithelial hyperplasia	0	5 (45.5)	1 (8.3)
	1	6 (54.5)	4 (33.3)
	2	0 (0.0)	7 (58.3)
Liver inflammatory cell infiltration	0	7 (63.6)	7 (63.6)
	1	4 (36.4)	4 (36.4)

**Liver fatty degeneration**	0	3 (27.3)	1 (8.3)
	1	4 (36.4)	3 (25.0)
	2	4 (36.4)	3 (25.0)
	3	0 (0.0)	5 (41.7)
